# Soluble sugar component-based quality assessment and SWEET transport family identification uncovering soluble sugar accumulation mechanism in *Zostera marina*

**DOI:** 10.3389/fpls.2026.1789397

**Published:** 2026-05-05

**Authors:** Meng Wu, YueChen Liu, HuiJiao Li, MengJiao Long, YaPing Gao, XianJun Fu, Lei Zhang

**Affiliations:** 1 Research Institute for Marine Traditional Chinese Medicine (Qingdao Academy of Chinese Medical Sciences), The State Administration of Traditional Chinese Medicine (SATCM) Key Unit of Discovering and Developing New Marine Traditional Chinese Medicine (TCM) Drugs, Key Laboratory of Marine Traditional Chinese Medicine in Shandong Universities, Shandong University of Traditional Chinese Medicine, Jinan, China; 2 State Key Laboratory of Mariculture Biobreeding and Sustainable Goods, Yellow Sea Fisheries Research Institute, Chinese Academy of Fishery Sciences, Qingdao, China

**Keywords:** bioinformatics, chemometrics, sugar transporter, SWEET, *Zostera marina*

## Abstract

*Zostera marina* L. is a marine medicinal plant with important ecological functions and considerable medicinal value. However, the absence of a systematic quality evaluation system has limited the development and utilization of its medicinal resources. In addition, the molecular basis of soluble sugar accumulation and transport in *Zostera marina* remains poorly understood. In this study, four Zosteraceae species were collected, and the contents of 11 soluble sugar components were determined. A quality evaluation system based on soluble sugar components was established using chemometric methods. Furthermore, based on the genomic data of *Zostera marina*, members of the SWEET gene family were systematically identified and analyzed for their physicochemical properties, subcellular localization, transmembrane domains, conserved motifs, gene structures, and cis-acting regulatory elements. The transport activity of selected ZosmaSWEET proteins was further verified in yeast. The results showed that *Zostera marina* had clear advantages in the contents of glucose, fructose, and several other soluble sugar components, indicating that it represents a high-quality medicinal germplasm resource. A total of eight ZosmaSWEET genes were identified in *Zostera marina*. Most of the encoded proteins were predicted to localize to the plasma membrane and contained typical transmembrane domains, conserved motifs, and domain architectures. Cis-acting element analysis suggested that the expression of these genes is mainly regulated by light signals and hormones. Among them, ZosmaSWEET8 exhibited glucose transport activity in yeast. This study established a soluble sugar component-based quality evaluation system for Zosteraceae species and systematically elucidated the structural and functional characteristics of the SWEET gene family in *Zostera marina* for the first time. These findings provide theoretical and technical support for the development, quality control, and molecular breeding of marine medicinal plant resources.

## 1 Introduction

Seagrass beds are typical nearshore marine ecosystems and possess important ecological functions. In China, seagrasses are represented by four families, namely Zosteraceae, *Hydrocharitaceae*, *Cymodoceaceae*, and *Ruppiaceae*. Among them, the *Zostera marina* L. (*Zostera marina*) a member of the family Zosteraceae, is the most widely distributed seagrass species in the Northern Hemisphere (Moore et al., 2025). In China, it is mainly distributed in the shallow coastal waters of Shandong, Hebei, and Liaoning Provinces*. In addition to* its crucial role in providing essential ecosystem services in shallow coastal environments (Iqbal et al., 2024; Kim et al., 2026; Nahirnick et al., 2019), *Zostera marina* has also been historically in traditional medicine. It was first recorded in the Bencao Shiyi, which described it as growing in the deep sea and in Silla, with leaves resembling those of aquatic algae but larger in size. Jiayou Bencao further documented its medicinal uses for inducing labor, treating gynecological disorders, wind-related ailments, and promoting diuresis. In addition, the Chinese Marine Materia Medica records that Zostera marina is used to treat edema and goiter, with therapeutic effects similar to those of Sargassum (Guan and Wang, 2009). These classical records indicate the long history of medicinal use of *Zostera marina*. Modern studies have shown that *Zostera marina* contains a variety of bioactive compounds, including polysaccharides, flavonoids, phenolics, organic acids, amino acids, and minerals (Sekiguchi et al., 2025; Elkattan et al., 2024; Huang, 2011). Pharmacological studies have further demonstrated that it possesses anti-inflammatory, antibacterial, anti-tubercular, antioxidant, anti-aging, and lipid-lowering properties, highlighting its considerable potential for pharmaceutical development (Aminina et al., 2021; Kim et al., 2015; Sun, 2014; Liu et al., 2010).

In the quality control of marine medicinal plants, polysaccharides are regarded as critical indicators for plant-derived marine herbal medicines. Fucose, glucose, and galactose are important soluble sugar constituents of fucoidan and are associated with immune regulation, anti-inflammatory activity, antioxidant effects, and the promotion of gut health. These compounds therefore have potential applications in antitumor therapy, skin protection, and the regulation of microbial balance. The 2025 edition of the Chinese Pharmacopoeia (Chinese Pharmacopoeia Commission, 2025) includes only two marine plant-derived medicinal materials: *Sargassum* and *Laminaria japonica*, for which fucose has been established as a primary quality control marker. This highlights the pivotal role of saccharide components in the quality assessment of marine medicinal plants. Chemometric techniques apply a range of mathematical and statistical approaches to extract meaningful chemical information from large datasets, comprehensively characterize sample quality, and provide high analytical specificity (Jiu et al., 2021). Through data reduction, pattern recognition, and quantitative modeling, chemometric methods can support the qualitative analysis, classification, and mechanistic interpretation of soluble sugar compounds. In wax apple germplasm fruits, characterization of soluble sugar and organic acid components combined with chemometric analysis revealed that fructose and glucose are the major contributors to sweetness (Zhang et al., 2025). This suggests that chemometric methods can effectively reveal the contribution of individual soluble sugar components, thereby providing a theoretical basis for germplasm evaluation, quality assessment, and breeding improvement. At present, research on *Zostera marina* is mainly focused on marine ecological restoration, while studies on its development as a medicinal resource and quality evaluation still face significant challenges. On the one hand, multiple Zosteraceae species coexist along the coastal waters of Shandong Province, China, and these species may differ markedly in morphology, ecological adaptability, and secondary metabolite accumulation. On the other hand, although the polysaccharides of *Zostera marina* are known to be complex polymers composed of soluble sugars such as glucose, mannose, fucose, and rhamnose (Tao, 2019), the specific composition, relative abundance, and structure-activity relationships of these polysaccharides remain unclear among different species. Therefore, establishing a standardized quality control system based on chemical composition would facilitate the screening of superior germplasm resources within Zosteraceae.

In plant cells, sugar transport is mainly mediated by proteins encoded by three gene families: sugar transporters (MSTs) (Slewinski, 2011), sucrose transporters (SUTs) (Kühn and Grof, 2010), and Sugars Will Eventually be Exported Transporters (SWEETs). Among these, MSTs and SUTs have been extensively studied. In 2010, a new class of sugar transport proteins, designated SWEETs, was identified. SWEET proteins possess bidirectional transport capacity and can facilitate the transmembrane movement of sugars along substrate concentration gradients (Chen, 2014). Previous studies have demonstrated that SWEET proteins play crucial regulatory roles in diverse physiological processes, including sugar transport, signal transduction, primary metabolism, and stress responses (Yang et al., 2025; Li et al., 2025; Song et al., 2024; Zhu et al., 2024). Initially identified in *Arabidopsis thaliana*, the SWEET gene family has since been characterized in numerous plant species, including *Oryza sativa* (Singh et al., 2024), *Glycine max* (Zheng et al., 2023), *Vitis vinifera* (Xia et al., 2023), and *Malus domestica* (Nie et al., 2022). These studies indicate that SWEET genes are broadly involved in plant growth, development, stress adaptation, and the regulation of sugar metabolism. However, current research on SWEET genes has been largely concentrated on terrestrial plants, and little is known about this gene family in marine medicinal plants. Further investigation of the specific functions and regulatory mechanisms of SWEET genes in marine medicinal species is therefore needed to better utilize these genetic resources and enhance the medicinal value of marine plant-derived medicines.

In this study, we systematically evaluated the quality of different Zosteraceae species distributed in the coastal waters of Shandong Province, China, using soluble sugar composition as a key chemical indicator to identify the most promising germplasm resources. Subsequently, based on the genomic data of *Zostera marina*, members of the SWEET gene family (designated ZosmaSWEETs) were systematically mined and identified, followed by comprehensive analyses of their phylogenetic relationships, gene structures, conserved motifs, cis-acting elements, and expression patterns under high-sugar conditions. Furthermore, the sugar transport capacities of the identified SWEET genes were functionally investigated, with particular emphasis on their ability to transport the major sugars present in *Zostera marina*. This integrated approach, combining chemotype-based germplasm screening with genomic and functional analyses of sugar transporters, not only provides important insights into the molecular mechanisms underlying soluble sugar accumulation and medicinal quality in marine plants, but also establishes a foundation for the targeted breeding, quality standardization, and sustainable utilization of *Zostera marina* as a marine-derived medicinal resource.

## 2 Materials and methods

### 2.1 Determination and analysis of soluble sugar component contents in Zosteraceae species

The four Zosteraceae species, *Zostera marina*, *Zostera japonica*, *Zostera caespitosa*, and *Phyllospadix iwatensis*, were collected from the coastal area of Rongcheng, Weihai, Shandong Province, China, which is an important distribution and conservation region for *Zostera marina*. Dried samples were ground into powder and passed through a 60-mesh sieve. For extraction, 0.1 g of each powdered sample was extracted twice with 10 mL of ultrapure water under ultrasonication at 50 °C for 60 min each time. The combined extracts were centrifuged at 8,000 rpm for 10 min at 4 °C, and the supernatant was collected and adjusted to 100 mL with ultrapure water. Proteins were removed from 1 mL of this extract by repeated treatment with 5 mL of Sevage reagent (n-butanol: chloroform=1:5, v/v) until no precipitate formed at the interface. For soluble sugar analysis, 1 mL of the deproteinized extract was hydrolyzed with 1 mL of 2 mol/L trifluoroacetic acid (TFA) at 100°C for 2 h in a sealed tube. The TFA was completely removed by evaporating the mixture to dryness under a nitrogen stream, followed by three cycles of reconstitution in methanol and re-evaporation. The residue was finally dissolved in 1 mL of ultrapure water, diluted 50-fold, filtered through a 0.22 μM nylon membrane, and analyzed by HPAEC-Q-Orbitrap HRMS. For chromatographic and mass spectrometric conditions: Separation was performed on a Thermo Scientific CarboPac PA20 analytical column (3.0 mm×150 mm) equipped with a corresponding guard column (3.0 mm×30 mm), maintained at 35°C. Isocratic elution was employed with a mobile phase ratio of 97.5:2.5 (v/v) (phase A: ultrapure water, phase B: 200 mmol/L NaOH), at a flow rate of 0.4 mL/min. The injection volume was 5 μL, and the total analysis duration was 20 min. Mass spectrometric detection utilized a heated electrospray ionization (HESI) source, operating in positive ion mode with full-scan data acquisition at a resolution of 70,000. Key ion source parameters were set as follows: ion source temperature 350°C, capillary temperature 320°C, sheath gas pressure 45 psi, auxiliary gas pressure 10 arb, and spray voltage 3.0 kV.

A comprehensive analysis of soluble sugar content across four Zosteraceae species was conducted. Performing significance analysis using the GraphPad Prism 8 software. The data were standardized and subjected to principal component analysis (PCA) with three-dimensional score plots generated using Origin 2024 software. Cluster analysis was performed based on Euclidean distance using Ward’s linkage method. In addition, an orthogonal partial least squares discriminant analysis (OPLS-DA) model was constructed using the SIMCA 13.0 software, and the importance of the projection variables (VIP) scores were calculated.

### 2.2 Phylogenetic analyses and classifications of the ZosmaSWEETs family members

Homologs of SWEET genes from different plant species were retrieved from the PLAZA public database (https://bioinformatics.psb.ugent.be/plaza/) (Jiang et al., 2024), and the phylogenetic distribution of this gene family was analyzed to infer its possible evolutionary origin. SWEET protein sequences from *Zostera marina*, *Arabidopsis thaliana*, *Oryza sativa*, and *Spirodela polyrhiza* were aligned using MUSCLE in MEGA 7.0 with default parameters. A phylogenetic tree was subsequently inferred using the neighbor-joining (NJ) method and branch support was evaluated by bootstrap analysis with 1000 replicates. The resulting tree was visualized as a circular phylogenetic tree and refined using the Chiplot online platform (https://www.chiplot.online/) (Xie et al., 2023), with bootstrap values shown at the internal nodes. BLASTP was used to align the protein sequences of *Zostera marina*, *Oryza sativa*, and *Spirodela polyrhiza* for identifying homologous sequences, and MCScanX was employed to screen interspecific syntenic gene pairs. Finally, the Synteny Plot module in TBtools was utilized to visualize the chromosomal or scaffold and syntenic gene pair relationships among the three species: chromosomes or scaffolds of different species are displayed as linear tracks, while homologous gene pairs are indicated by connecting lines.

### 2.3 Identification and sequence analysis of ZosmaSWEETs

The whole-genome sequence and gene annotation file of *Zostera marina* (version v2.2) were obtained from the Phytozome v13 database (https://phytozome-next.jgi.doe.gov/) (Zhang et al., 2020). Candidate ZosmaSWEETs proteins were identified using the Simple HMM Search tool in TBtools-II (https://github.com/CJ-Chen/TBtools-II/releases) (Yang et al., 2023) with the PF03083 HMM profile, and sequences with an E-value<1 were retained for further analysis. The basic physicochemical properties of the identified ZosmaSWEETs proteins, including the number of amino acids, molecular weight (MW), theoretical isoelectric point (pI), and grand average of hydrophobicity (GRAVY), were calculated using the ProtParam tool (https://web.expasy.org/protparam/) (Muccee et al., 2024). Subcellular localization was predicted using the WoLF PSORT online server (https://wolfpsort.hgc.jp/) (Kumar et al., 2023). Transmembrane helices were predicted and visualized using the TMHMM-2.0 server (https://services.healthtech.dtu.dk/services/TMHMM-2.0/) (Parveen et al., 2025).

To examine gene structure, the coding sequences and corresponding genomic DNA sequences of the identified ZosmaSWEETs were submitted to the GSDS 2.0 server for visualization of exon-intron organization (Ling et al., 2024). Conserved protein domains were identified using the Conserved Domain Database (CDD) tool (Jiang et al., 2022). Conserved motifs were analyzed using MEME version 5.5.4 (Bailey et al., 2015). Finally, to predict the three-dimensional structures of ZosmaSWEET proteins, their amino acid sequences were submitted to the SWISS-MODEL server for exploratory structural modeling based on template-dependent homology modeling, using structural references derived from either experimentally predicted models from the AlphaFold database, with the corresponding identifiers provided consistently in the manuscript and supplementary **Supplementary Table 1** (https://swissmodel.expasy.org/) (Jiang et al., 2023).

### 2.4 Cis-regulatory element and expression profiling of ZosmaSWEETs

To identify potential cis-regulatory elements in the promoters of ZosmaSWEETs genes, a 2 kb genomic region upstream of the transcription start site of each gene was extracted and analyzed using the PlantCARE database (http://bioinformatics.psb.ugent.be/webtools/plantcare/html/) (Lescot et al., 2002). The distribution of predicted cis-acting elements was visualized with TBtools. To investigate the expression patterns of ZosmaSWEETs across different tissues of *Zostera marina*, a publicly available transcriptomic dataset (Accession: GSE67579) was downloaded from the NCBI database. The dataset includes five tissue types: early-stage female flowers, late-stage female flowers, male flowers, roots, and vegetative shoots. The mean expression levels of ZosmaSWEETs in each tissue were log_2_-transformed, and a heatmap was generated to visualize their tissue-specific expression profiles.

The ZosmaSWEETs were uploaded to the STRING database to construct a protein-protein interaction network of common target proteins. The network data were exported in TSV format and imported into Cytoscape 3.10.1. Topological analysis of the network was performed using the “Network Analyzer” module. In the resulting network graph, node size and color were arranged according to “degree”, and edge thickness was arranged according to the “combined score”.

### 2.5 Sugar transport activity assay and molecular docking of ZosmaSWEETs

To validate the sugar transport activity of ZosmaSWEETs, target gene fragments were amplified by polymerase chain reaction (PCR). For sequence synthesized of primers, the services of Sangon Biotech (Shanghai, China) were utilized. The pDR196 vector was digested with two restriction enzymes (**Supplementary Table 2**). The PCR product was ligated into the linearized vector using OK Clon DNA Ligation Kit (AG11803) purchased from ACCURATE BIOTECHNOLOGY (HUNAN) CO., LTD, ChangSha, China. After sequence verification, the recombinant plasmids were transformed into the hexose transporter-deficient yeast strain EBY.VW4000 (Ye et al., 2023) using the lithium acetate method. Transformed yeast cells were plated on SC-Ura medium supplemented with different types of sugars and incubated statically at 30°C for 3–5 days. Colony growth was monitored to visualize sugar transport ability.

To further investigate the molecular interactions between ZosmaSWEETs and glucose, the three-dimensional protein structures of ZosmaSWEETs were first modeled using SWISS-MODEL. Protein files were prepared using AutoDock Tools. The structural quality and stereochemical rationality of the predicted protein models were evaluated using the Ramachandran plot generated on the UCLA-DOE SAVES platform (https://saves.mbi.ucla.edu/). The molecular structure of glucose, fructose, and sucrose were retrieved from the PubChem database. Protein and ligand files were prepared with AutoDock Tools (Ma et al., 2024). The docking grid was centered on the predicted active pocket of the protein, with grid box coordinates of x=0.414, y=10.934, and z=12.162. The resulting protein-glucose binding poses were visualized using PyMOL (Seeliger and de Groot, 2010).

## 3 Results

### 3.1 Morphological differences and selection of superior germplasm in Zosteraceae

**Figure 1:**
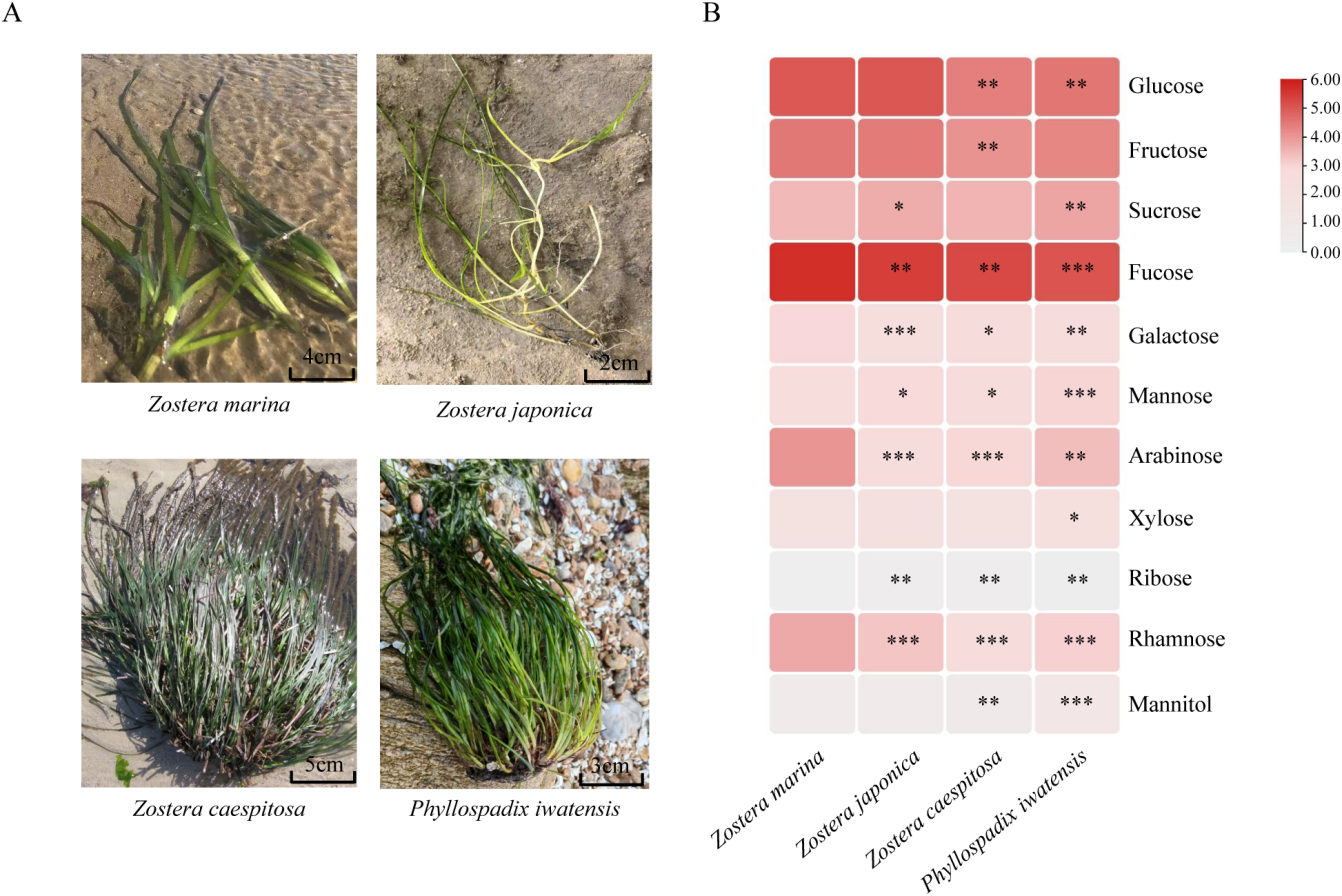
Detection of morphology and content in different varieties of Zosteraceae species. **(A)** Phenotypes of different Zosteraceae species (*Zostera caespitosa*, *Zostera japonica*, *Zostera marina*, *Phyllospadix iwatensis*). **(B)** Heat map of the contents of 11 soluble sugar components in different Zosteraceae species, with *Zostera marina* as the control group (n=3). All data were subjected to log2 transformation. Asterisks indicate statistical significance (*P < 0.05, **P < 0.005, and ***P < 0.0005).

Morphological observations of the four Zosteraceae species revealed clear interspecific differences. *Zostera marina* showed a loose growth habit characterized by creeping rhizomes, with broad, long, and relatively tough leaves. *Zostera japonica* was smaller and more slender, with slender rhizomes (diameter < 0.5-1.5 mm) and thin, short, yellowish-green leaves. *Zostera caespitosa* exhibited a densely caespitose growth habit, with extremely short or suberect rhizomes and tightly arranged basal sheaths. *Phyllospadix iwatensis* displayed a compact growth habit with clustered leaves, thick and short rhizomes, and reddish-brown fibrous material surrounding the rhizome bases. Its leaves were linear, bright green, and rigid in texture. (**Figure 1A**). The heatmap (**Figure 1B**) revealed distinct differences in soluble sugar composition among the examined Zosteraceae species, with *Zostera marina* used as the control. Glucose, fructose, and especially fucose were the dominant soluble sugars, whereas ribose and mannitol were present at relatively low levels in *Zostera marina*. Relative to the control, *Zostera japonica*, *Zostera caespitosa*, and *Phyllospadix iwatensis* generally showed lower levels of fucose, galactose, arabinose, and rhamnose, with several of these differences reaching statistical significance. In contrast, ribose was significantly increased in all three non-control species, while mannitol was significantly elevated in *Zostera caespitosa* and *Phyllospadix iwatensis*. Sucrose was significantly higher in *Zostera japonica* and *Phyllospadix iwatensis*, and glucose was significantly reduced in *Zostera caespitosa* and *Phyllospadix iwatensis*. This indicates significant differences in soluble sugar composition among different Zosteraceae species.

**Figure 2:**
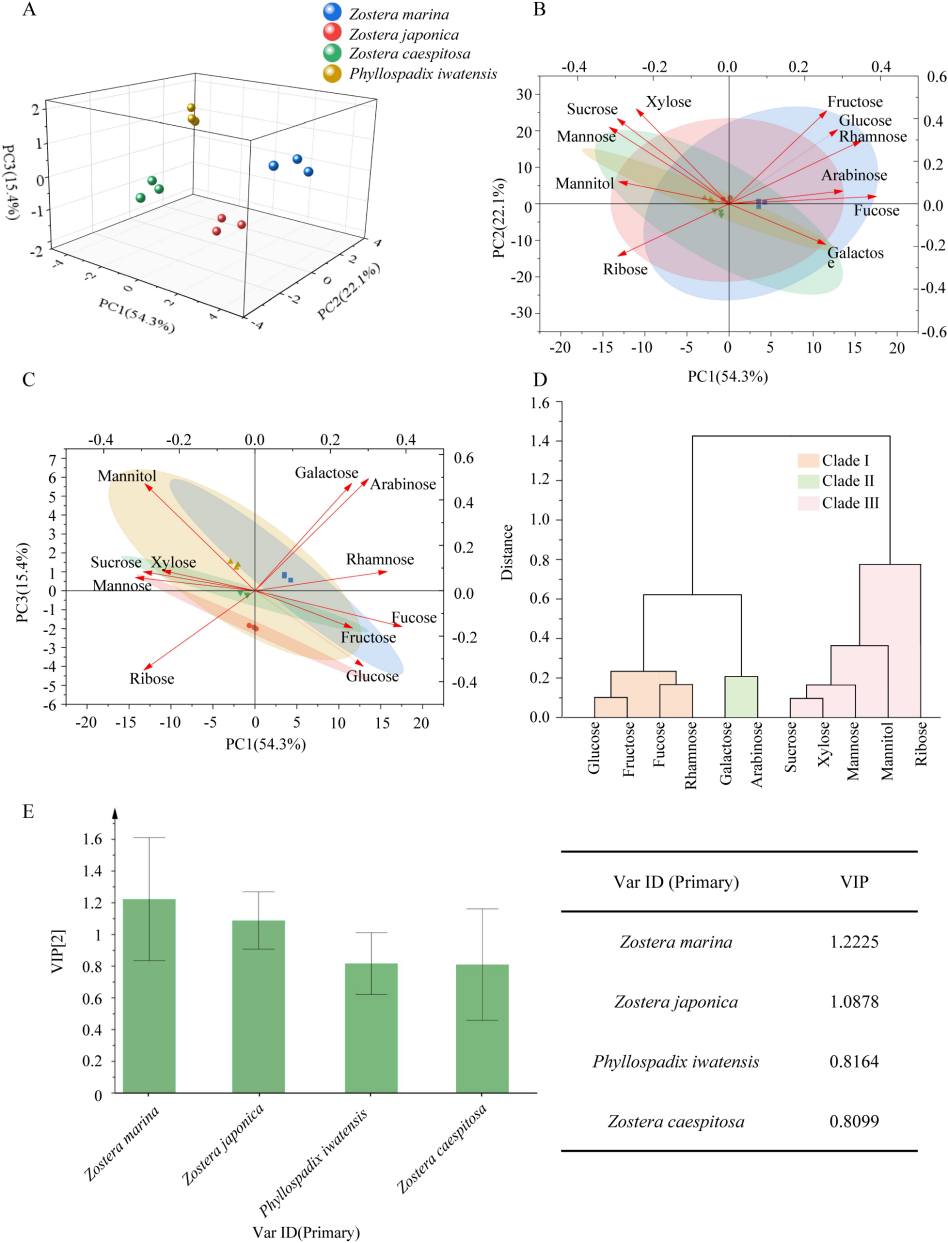
Soluble sugar component content analysis in different Zosteraceae species. **(A)** Three-dimensional score plot from Principal Component Analysis (PCA) of soluble sugar components across different Zosteraceae species. **(B)** PCA score plot (PC1 vs. PC2) for soluble sugar components in different Zosteraceae species. **(C)** PCA score plot (PC1 vs. PC3) for soluble sugar components in different Zosteraceae species. **(D)** Cluster analysis results for soluble sugar components in different Zosteraceae species. **(E)** Variable Importance in Projection (VIP) score plot for different Zosteraceae species.

In order to further analyze the quality of different varieties of Zosteraceae, chemometric analysis was conducted. Principal component analysis (PCA) and orthogonal partial least squares discriminant analysis (OPLS-DA) were subsequently performed based on the soluble sugar content data (**Figure 2**). In the three-dimensional PCA score plot (**Figure 2A**), the sample size for each species was three biological replicates (n = 3), and the explained variance ratios for PC1, PC2, and PC3 were 54.3%, 22.1%, and 15.4%, respectively. The differently colored points represent the four species (*Zostera marina*, *Zostera japonica*, *Phyllospadix iwatensis*, and *Zostera caespitosa*), which showed clear group separation in the three-dimensional space, indicating marked species-specific differences in their compositional profiles. The PCA loading plot, explaining approximately 91.8% of the total variance, provides a more comprehensive visualization of how individual components contribute to the separation of species clusters. The arrows representing components such as fucose and glucose point towards the *Zostera marina* cluster, indicating that these compounds are the primary factors driving its separation along the PC1-PC2 dimensions (**Figure 2B**). The arrows for mannitol and xylose are oriented more towards *Zostera japonica*, suggesting that these components play a key role in distinguishing this species from others in the PC1-PC3 dimensional space (**Figure 2C**). Hierarchical cluster analysis (HCA) of sugar components across different varieties (**Figure 2D**) grouped the 11 soluble sugar compounds into three distinct clusters: Cluster I primarily included glucose, fructose, fucose, and rhamnose. Cluster II mainly consisted of galactose and arabinose. Cluster III was predominantly composed of sucrose, xylose, mannose, mannitol, and ribose. We observed that the soluble sugar components in Cluster I exhibited moderate to high contents, while those in Clusters II and III showed relatively low contents. Additionally, soluble sugar with more similar content levels displayed closer clustering distances. In the OPLS-DA model, variable importance in projection (VIP) values were calculated, where a VIP value > 1 typically indicates that the variable is a key contributor to discriminating between sample groups. The results showed that *Zostera marina* had the highest VIP value (1.2225), followed by *Zostera japonica* (1.0878), suggesting that these two species are the most distinctive and potentially high-quality varieties within the Zosteraceae.

### 3.2 Species evolution analysis based on single-copy direct homologous genes

**Figure 3:**
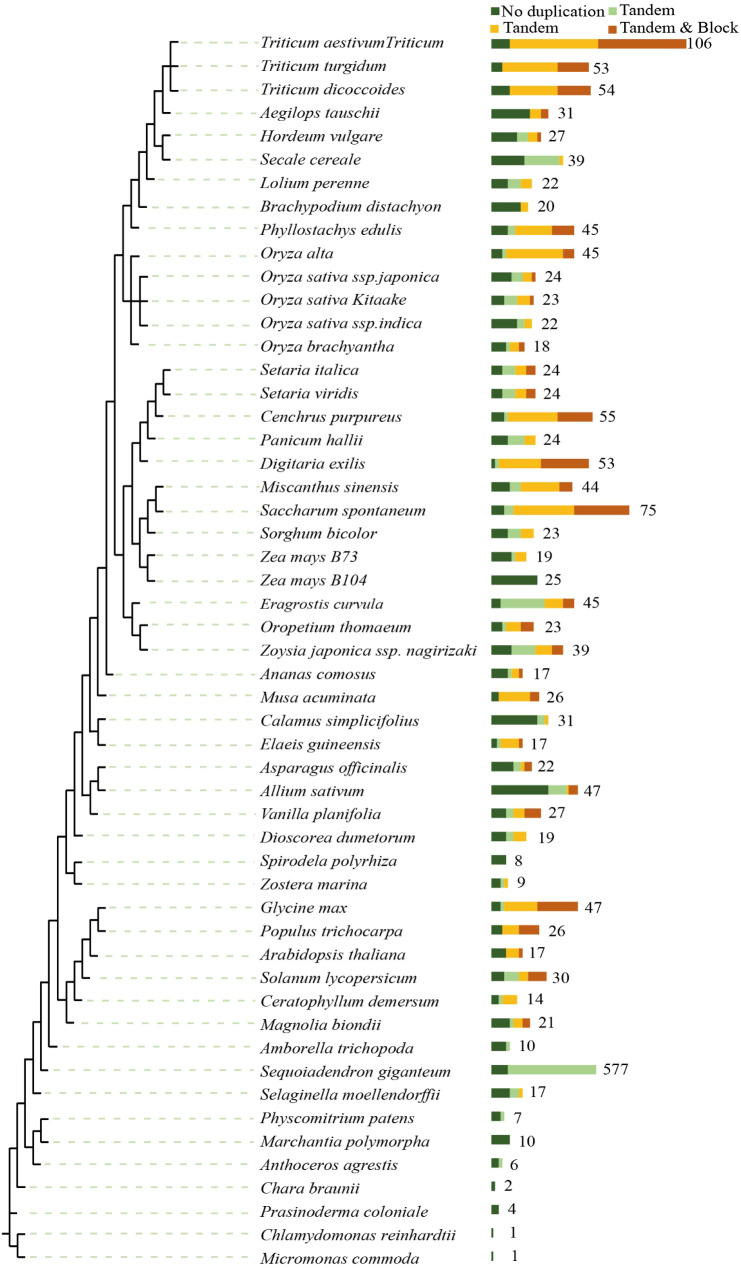
Phylogenetic tree construction of plant species based on the ZosmaSWEET gene family in *Zostera marina*.

SWEET proteins can specifically transport glucose, fructose, galactose, or sucrose in different plant species (Yin et al., 2020). Because *Zostera marina* contains 11 types of soluble sugars, the SWEET gene family represents an important target for exploring the molecular mechanisms of sugar transport in this species. In this study, we identified ZosmaSWEET genes and performed phylogenetic analyses to clarify the origin and evolutionary history of this gene family. A total of 1,495 homologous genes were identified across 53 plant species (**Figure 3**). The proportions of tandem, block, and combined duplication events were 17%, 32%, and 20%, respectively, suggesting that the SWEET gene family has undergone extensive duplication during evolution. *Zostera marina* clustered within the monocot lineage and showed closer phylogenetic relationships with other monocots, such as *Asparagus officinalis* and *Allium sativum*, as well as with the early-diverging lineage *Spirodela polyrhiza*. Furthermore, gene duplication analysis revealed substantial variation among species. Notably, only two duplication events were detected in *Zostera marina*. This relatively limited expansion of the ZosmaSWEET gene family may be associated with reduced gene family expansion, fewer duplication events, and weaker selective pressure under the relatively stable marine environment.

### 3.3 Evolutionary and molecular characterization of ZosmaSWEETs in *Zostera marina*

**Figure 4:**
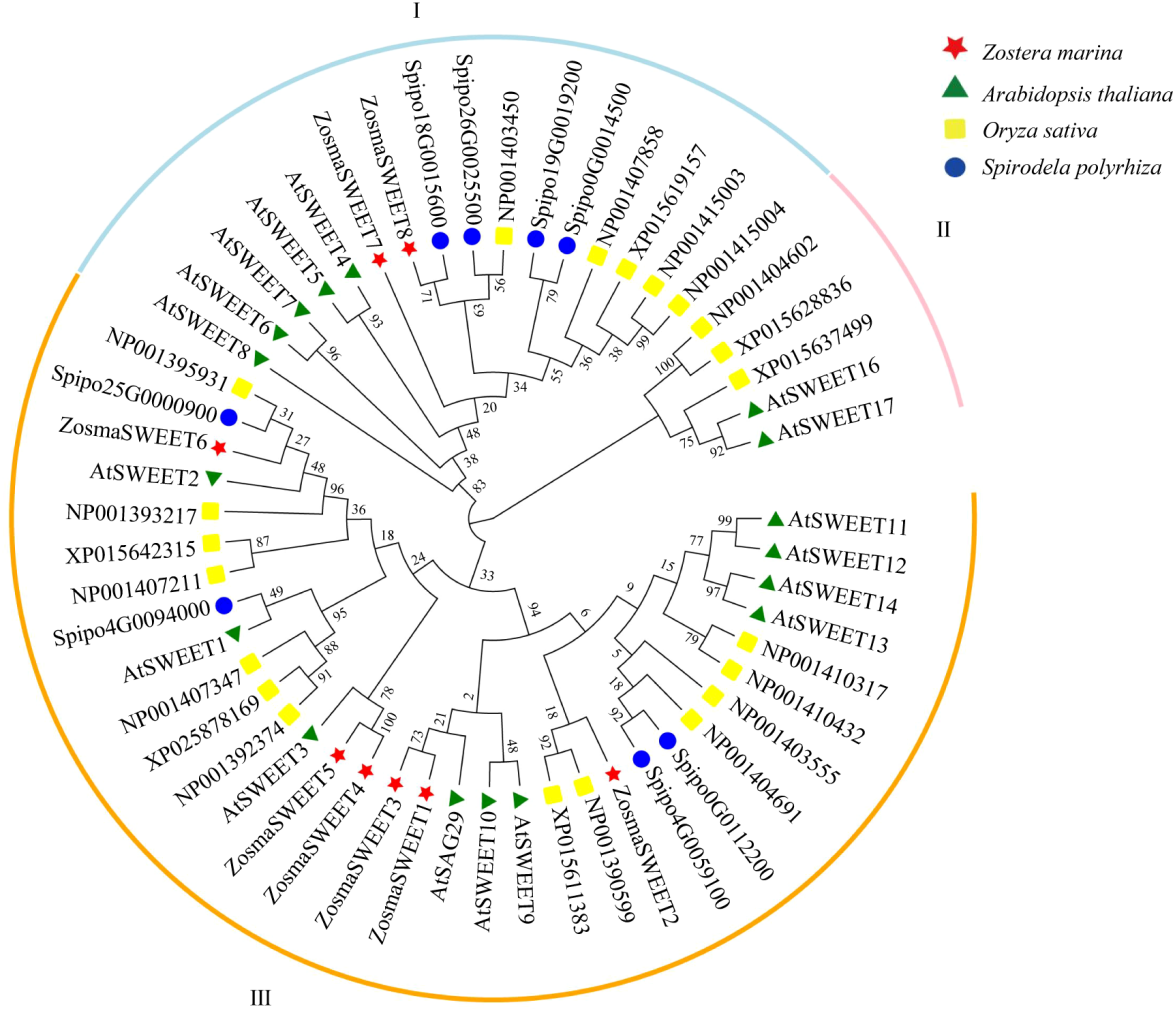
Phylogenetic tree for SWEET proteins identified from *Zostera marina* (red star), *Arabidopsis thaliana* (green triangle), *Oryza sativa* (yellow square), and *Spirodela polyrhiza* (blue circle).

Construction and analysis of phylogenetic trees provide an important basis for clarifying taxonomic relationships, tracing evolutionary origins, and predicting gene functions in non-model plants. Based on the genome of *Zostera marina*, eight non-redundant ZosmaSWEET members were identified. A phylogenetic tree was constructed using SWEET proteins from *Arabidopsis thaliana*, *Oryza sativa*, and *Spirodela polyrhiza* (**Figure 4**). The tree was divided into three major clades (Clades I, II, and III), and genes within the same clade exhibited closer evolutionary relationships. The ZosmaSWEET genes did not form an independent cluster, but were distributed among different subfamilies together with genes from monocots, such as *O. sativa*, and early-diverging angiosperms, such as *S. polyrhiza*. This distribution pattern suggests that the SWEET gene family in *Zostera marina* has undergone a complex evolutionary history involving both retention of ancestral genes and lineage-specific divergence. These results indicate that *Zostera marina* has retained ancient gene lineages shared with both terrestrial and aquatic plants, while also potentially developing lineage-specific branches associated with environmental adaptation. This phylogenetic framework provides an evolutionary basis for further investigation of the regulatory mechanisms underlying sugar transport in marine environments. Collinearity analysis of SWEET genes among *O. sativa*, *Zostera marina*, and *S. polyrhiza* (**Supplementary Figure 1**) revealed no direct syntenic relationship between *O. sativa* and *Zostera marina*. In contrast, clear collinear relationships were detected between *S. polyrhiza* and *Zostera marina*. Specifically, Spipo4G0059100 in *S. polyrhiza* showed direct collinearity with *Zosma7g01420* located on scaffold_7 of *Zostera marina*, while *Spipo26G0025500* corresponded collinearly with *Zosma425g00100* on scaffold_425. These findings suggest that these gene pairs likely originated from common ancestral genes and have retained chromosomal synteny during the evolution of *S. polyrhiza* and *Zostera marina*, reflecting a relatively high degree of evolutionary conservation.

A total of eight ZosmaSWEET genes were identified in *Zostera marina*, and their physicochemical properties and subcellular localizations were further analyzed (**Table 1**). The protein lengths of these members showed relatively limited variation, with predicted molecular weights ranging from 26.2 to 48.9 kDa. The isoelectric points of all members were greater than 7, indicating that these proteins are alkaline in nature. Among them, ZosmaSWEET2, ZosmaSWEET3, and ZosmaSWEET6 exhibited GRAVY values greater than 0.8, suggesting strong hydrophobicity, and their instability indices were generally below 40, indicating relatively high stability. In contrast, ZosmaSWEET1 had a GRAVY value of -0.222, indicating hydrophilicity, and its instability index was slightly higher than 40, suggesting relatively lower stability. Subcellular localization prediction revealed clear differences among ZosmaSWEET family members. Most proteins, including ZosmaSWEET1, ZosmaSWEET2, ZosmaSWEET3, ZosmaSWEET5, and ZosmaSWEET8, were predicted to localize to the plasma membrane, consistent with their potential roles in transmembrane transport. ZosmaSWEET4 was predicted to localize to the endoplasmic reticulum, suggesting a possible role in intracellular protein processing or transport. In contrast, ZosmaSWEET6 and ZosmaSWEET7 were predicted to localize to the vacuole, indicating potential functions in vacuolar transport and intracellular substance sequestration. Overall, although the physicochemical properties of ZosmaSWEET family members are relatively conserved, their distinct subcellular localization patterns suggest functional diversification in processes such as sugar transport and cellular metabolism.

**Table 1:** The protein length, molecular weight, pI, GRAVY, instability index, and subcellular localization of ZosmaSWEETs.

Gene ID	Putative gene name	Protein length (AA)	MW (Da)	pI	GRAVY	Instability index	Subcellular localization
Zosma3g01130	ZosmaSWEET1	427	48879.37	9.89	-0.222	40.57	Plasma membrane
Zosma7g01420	ZosmaSWEET2	293	32627.95	7.66	0.893	33.40	Plasma membrane
Zosma23g00230	ZosmaSWEET3	249	27599.88	9.23	0.876	35.23	Plasma membrane
Zosma34g00490	ZosmaSWEET4	232	26274.70	9.55	0.600	41.85	Endoplasmic reticulum
Zosma34g00500	ZosmaSWEET5	250	27657.13	9.56	0.637	40.91	Plasma membrane
Zosma70g00350	ZosmaSWEET6	232	26235.31	9.01	0.943	40.32	vacuole
Zosma376g00060	ZosmaSWEET7	236	26419.65	9.46	0.609	44.19	vacuole
Zosma425g00100	ZosmaSWEET8	255	28182.79	9.21	0.720	39.04	Plasma membrane

### 3.4 Gene structure and conserved protein motif analysis of ZosmaSWEETs

**Figure 5:**
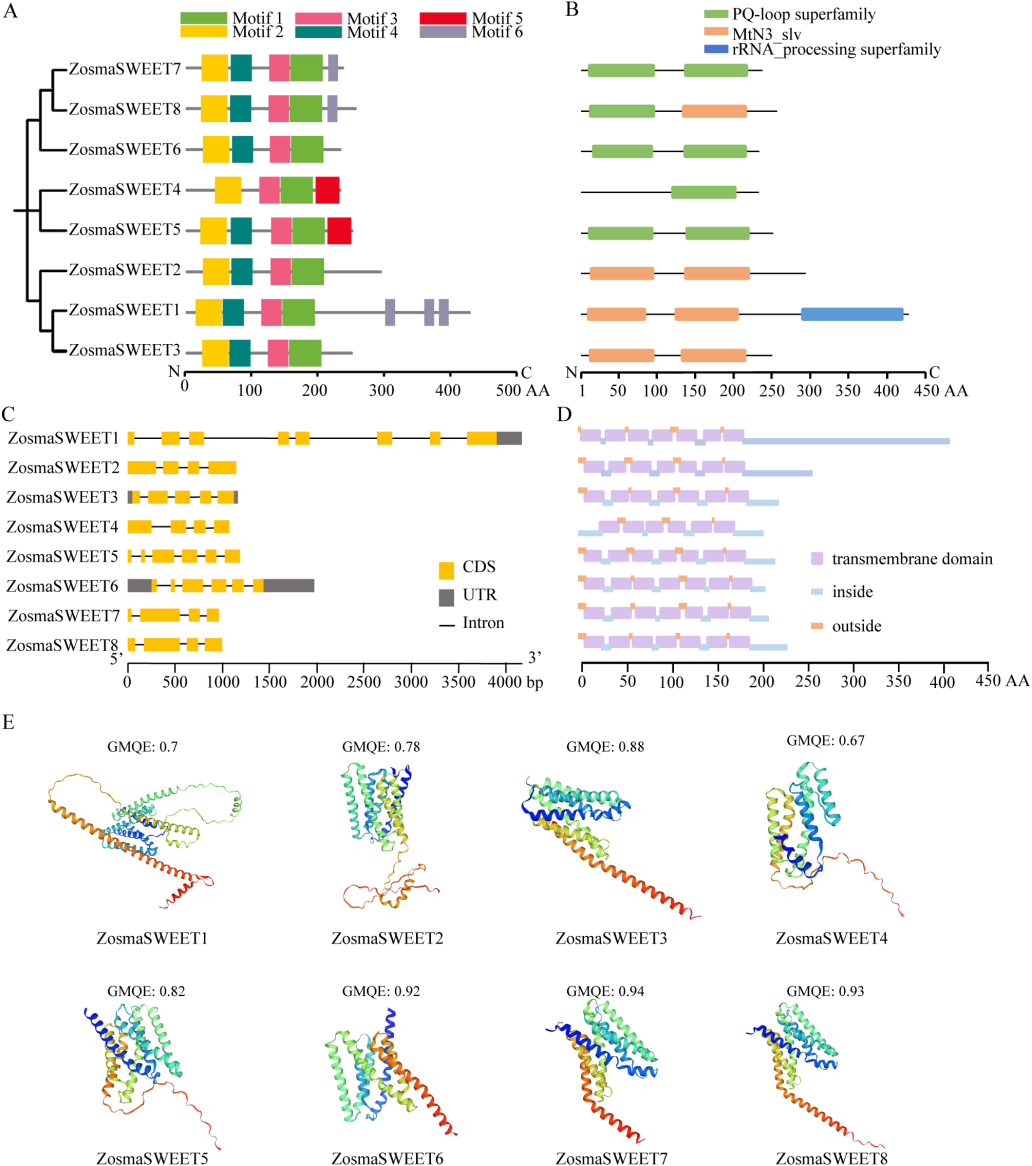
Identification and analysis of ZosmaSWEET genes in *Zostera marina*. **(A)** Conserved motifs of ZosmaSWEET proteins, including 6 motifs, **(B)** Conserved domains of ZosmaSWEET proteins, **(C)** Exon/intron structures of ZosmaSWEET genes. The yellow color was the untranslated region (UTR), while the gray was the coding sequence. The line without color referred to introns, **(D)** Protein transmembrane structures of ZosmaSWEET proteins. The blue line indicates the inside of the cell membrane, the orange line indicates the outside of the cell membrane, and the purple box indicates transmembrane domain, **(E)** Three-dimensional structures of ZosmaSWEET proteins predicted using the SWISS-MODEL.

A combined analysis of gene structure and conserved protein motifs was performed for all identified ZosmaSWEET genes to clarify their evolutionary conservation and subfamily characteristics. MEME analysis was conducted to characterize the composition, distribution, and sequence features of conserved motifs in *ZosmaSWEET1*-*ZosmaSWEET8* (**Figure 5A**). The results identified six conserved motifs (Motifs 1-6) in the ZosmaSWEET family. All members contained Motif 1, Motif 2, and Motif 3, indicating a relatively high degree of conservation within the family. Motif 5 was detected only in *ZosmaSWEET4* and *ZosmaSWEET5*. Examination of the motif logos (**Supplementary Figure 2**) further showed that Motif 5 is highly conserved, suggesting that it may represent a characteristic conserved motif in specific ZosmaSWEET members. Conserved domain analysis identified three structural domains in the ZosmaSWEET proteins (**Figure 5B**). The PQ-loop superfamily domain, labeled in green, was present in *ZosmaSWEET4-8*, whereas the MtN3_slv domain, labeled in orange, was detected in *ZosmaSWEET1-3*. In addition, only *ZosmaSWEET1* contained the rRNA-processing superfamily domain, labeled in blue. These results suggest structural divergence among ZosmaSWEET members, which may be associated with functional differentiation. Gene structure analysis showed that all ZosmaSWEET genes contained multiple exons and introns (**Figure 5C**), although untranslated regions were not detected in all members. Among them, ZosmaSWEET1 contained the highest number of coding sequences. Transmembrane structure prediction indicated that all ZosmaSWEET proteins possess multiple transmembrane domains (highlighted in purple), and most exhibited a typical multi-pass transmembrane topology (**Figure 5D**). ZosmaSWEET6 contained six transmembrane domains, whereas all other members contained seven. Three-dimensional protein structure modeling further demonstrated that all ZosmaSWEET family members adopt a typical transmembrane protein conformation, with α-helices and β-sheets forming the major secondary structural framework (**Figure 5E**).

### 3.5 Promoter cis-acting elements and expression patterns analysis of ZosmaSWEETs

**Figure 6:**
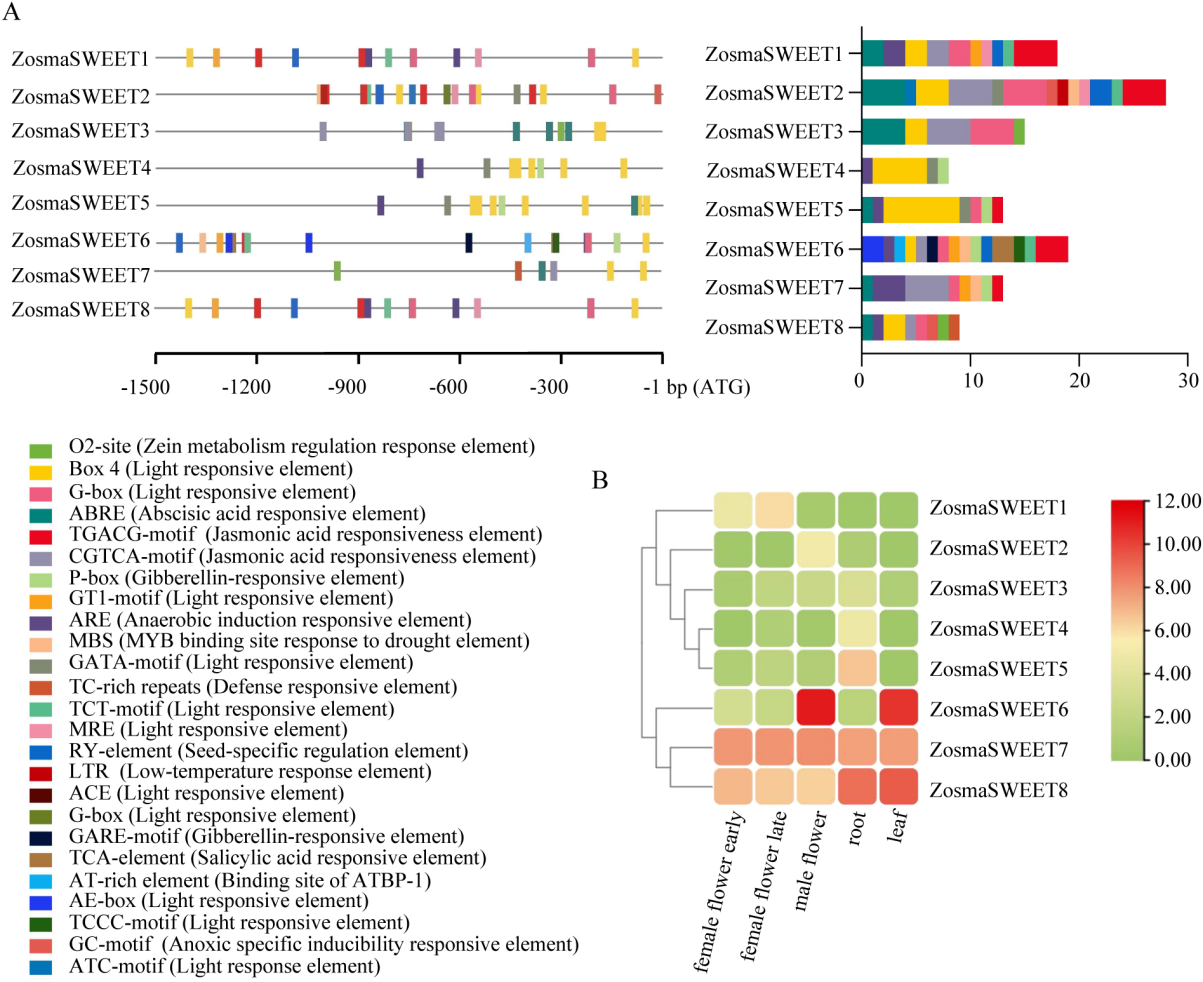
Analysis of cis-regulatory elements and expression patterns of the SWEET gene family in *Zostera marina*. **(A)** Cis-element analysis of ZosmaSWEETs promoters related to responses. The right column chart is a diagram showing the statistical condition of cis-acting elements. Cis-elements with functional similarity are shown in the same color. **(B)** Expression profiles of ZosmaSWEETs in different samples. All data were subjected to log2 transformation.

Analysis of cis-acting elements in the promoters of ZosmaSWEET genes provides important insight into the regulatory patterns and potential functions of this gene family, thereby establishing a basis for investigating the expression profiles and environmental adaptation mechanisms of *Zostera marina*. A total of 25 categories of cis-acting elements related to abiotic stress responsiveness, hormone signaling, and growth and development were identified within the 2,000 bp promoter regions upstream of the start codons of ZosmaSWEET genes (**Figure 6A**). Among these elements, light-responsive elements accounted for the largest proportion (41.46%), with Box 4 and GT1-motif being particularly abundant. Hormone-responsive elements represented 39.84% of the total, among which abscisic acid-responsive and jasmonic acid-responsive elements were the most prevalent. Stress-responsive elements accounted for the smallest proportion (17.07%) and included anaerobic induction elements, seed-specific regulatory elements, and MYB-binding sites involved in drought responsiveness. The heatmap of ZosmaSWEET expression patterns across different tissues revealed substantial differences among family members (**Figure 6B**). ZosmaSWEET1–5 generally exhibited low expression levels in the tissues examined. In contrast, ZosmaSWEET6 showed relatively high expression in male flowers and leaves, whereas ZosmaSWEET7 displayed similar expression levels across different tissues. ZosmaSWEET8 was highly expressed in roots and leaves. These results suggest that ZosmaSWEET6–8 may play important roles in sugar transport in *Zostera marina*. Screening of proteins interacting with ZosmaSWEETs further revealed that Zosma22g01450, Zosma1g00550, and Zosma31g00920 had the highest degree values, indicating the largest numbers of interaction connections within the network (**Supplementary Figure 3**). This finding suggests that these three proteins may function as core hub proteins in the ZosmaSWEET interaction network, potentially participating in signal transduction or material transport pathways during different physiological processes through interactions with multiple ZosmaSWEET members.

### 3.6 ZosmaSWEET8 has the ability to transport fructose, glucose, and sucrose

**Figure 7:**
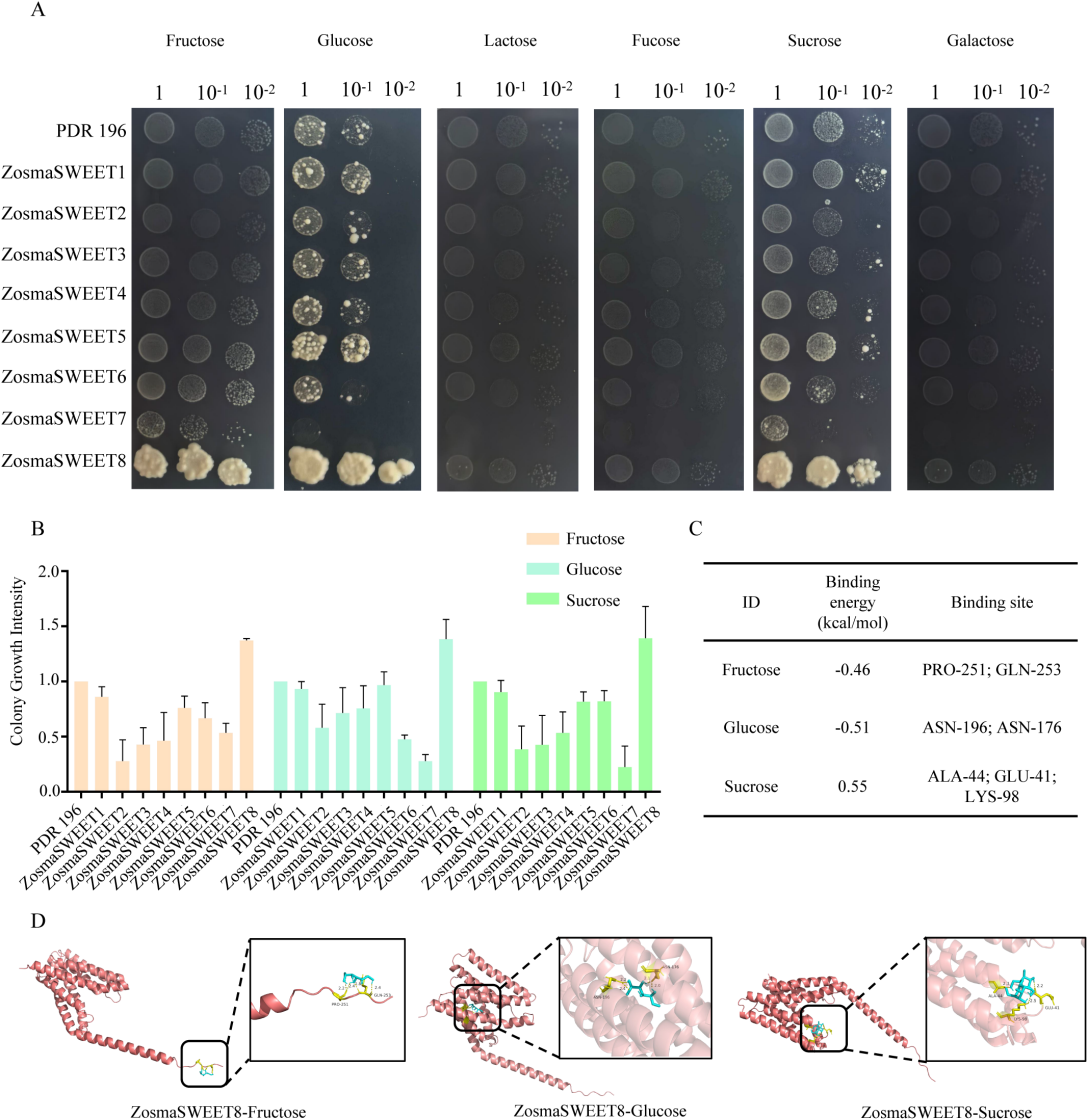
Sugar transport activity of the ZosmaSWEET gene family. **(A)**. Growth of yeast mutant strain EBY. VW4000 expressing different genes in SC/-Ura medium. **(B)** The growth situation of the sweet gene family in the SC/-Ura medium containing 2% fructose, 2% glucose and 2% sucrose. **(C)** The molecular docking binding energy and binding site information in ZosmaSWEET8. **(D)** Visualization of molecular docking of ZosmaSWEET8 with different sugars.

Bioinformatic analysis clarifies structural characteristics and functional potential of ZosmaSWEET genes, critical for studying their sugar transport functions. Therefore, we cloned the CDS of ZosmaSWEETs into the PDR196 vector and successfully transformed it into the sucrose transport-deficient mutant EBY.VW4000 to evaluate its activity and explore the sugar transport function of ZosmaSWEETs. (**Figures 7A, B**). The results indicated differential sugar transport activities of ZosmaSWEETs under different carbon source treatments. Notably, the EBY.VW4000 yeast strain carrying *ZosmaSWEET8* grew normally on media containing 2% glucose, fructose, or sucrose, demonstrating that *ZosmaSWEET8* can specifically transport glucose into yeast cells. Furthermore, the sugar-binding capability of *ZosmaSWEET8* was verified at the molecular level (**Figures 7C, D**). The Ramachandran plot analysis showed that 96.5% of the residues in ZosmaSWEET8 were located in the core region, indicating that the predicted model possessed good overall stereochemical quality and was suitable for subsequent molecular docking analysis (**Supplementary Table 1**). Molecular docking data revealed that *ZosmaSWEET8* exhibited negative binding energies with fructose and glucose, indicating stable binding interactions, while its binding energy with sucrose was positive, suggesting weaker binding affinity. Three-dimensional binding models visually illustrated the binding sites of *ZosmaSWEET8* with different sugars, providing direction for further research.

## 4 Discussion

In this study, we first screened high-quality *Zostera marina* germplasm with promising medicinal potential from a chemometric perspective, and further characterized the evolutionary, structural, and functional features of the ZosmaSWEET sugar transporter family using molecular biological approaches. These findings provide a new perspective for understanding the formation of medicinal quality in seagrasses and the regulation of sugar metabolism in marine plants. Significant species-specific differences in soluble sugar composition were observed among the different seagrass species (**Figure 2**). In particular, *Zostera marina* exhibited the highest VIP value, highlighting its distinctive chemical characteristics and supporting its classification as a superior medicinal germplasm resource within the genus. Consistent with previous reports identifying *Zostera marina* as a high-quality germplasm resource (Qiao et al., 2022), our findings provide additional chemometric evidence to support this conclusion. Furthermore, this study establishes a sugar composition-based quality evaluation framework, which may provide a useful reference for the future inclusion and quality assessment of marine medicinal plants in subsequent editions of the *Chinese Pharmacopoeia*.

The SWEET gene family, an important class of sugar transporter proteins, plays essential roles in physiological processes such as sugar transport, distribution, and storage in plants (Anjali et al., 2020; Jeena et al., 2019). Reported numbers of SWEET genes in higher plants range from 17 to 108. For instance, 17 SWEET genes have been identified in *Arabidopsis thaliana* (Chen et al., 2010), 108 in *Triticum aestivum* (Gautam et al., 2019), 23 in *Sorghum bicolor* (Mizuno et al., 2016), and 29 in *Solanum lycopersicum* (Feng et al., 2015). In this study, eight ZosmaSWEET members were identified in *Zostera marina*. These genes belong to the monocot lineage and show close evolutionary relationships with terrestrial monocots, although their number is markedly lower than that reported in most terrestrial plants. In addition, some members exhibited chromosomal synteny with homologous genes in *Spirodela polyrhiza*. The relatively small number of duplication events detected in *Zostera marina*, compared with terrestrial crops, further suggests that the sugar transport system in this species has remained relatively conserved during adaptation to the marine environment, where selective pressure for extensive gene family expansion may have been reduced. The MtN3_slv domain is a characteristic feature of SWEET family proteins and is generally associated with sugar transport activity (Liu et al., 2012). Conserved domain analysis (**Figure 5B**) showed that ZosmaSWEET proteins contain the characteristic MtN3_slv domain, supporting their classification as SWEET family members. Conserved motif analysis (**Figure 5A**; **Supplementary Figure 2**) further demonstrated that genes located in closely related phylogenetic branches tend to share similar motif compositions. At the same time, individual members within each branch also possessed distinct motif features, suggesting potential functional divergence among ZosmaSWEET proteins. Gene structure analysis revealed that ZosmaSWEET genes contain 4–7 introns. Similar exon-intron organization has also been reported in cucumber (Hu et al., 2017), soybean (Patil et al., 2015), and banana (Miao et al., 2017), indicating that SWEET family members are structurally conserved during evolution. These structural characteristics may influence gene expression patterns and protein functions, thereby contributing to sugar distribution and storage in *Zostera marina*. Cis-regulatory elements are a class of DNA sequences located in the transcription initiation region of genes, corresponding transcription factors recognize these cis-acting elements to regulate the mRNA abundance of target genes, playing a crucial role in abiotic stress responses (Liu et al., 2014). In this study, the predominance of light-responsive and hormone-responsive cis-acting elements in the promoters of ZosmaSWEET genes suggests that their expression may be mainly regulated by light signals and plant hormones, thus providing a foundation for future studies on how these regulatory pathways influence ZosmaSWEET expression, sugar transport, and sugar accumulation in *Zostera marina*. Collectively, these findings deepen our understanding of the evolutionary patterns and potential regulatory mechanisms of the SWEET gene family in non-model *Zostera marina*, and provide a framework for further investigation of the molecular mechanisms of sugar transport that are critical for seagrass survival and reproduction.

The SWEET gene family is crucial for regulating sugar accumulation in plants. Studies have shown that in *Sorghum dochna*, the SdSWEET01, SdSWEET06 and SdSWEET09 proteins are capable of transporting glucose (Pan et al., 2024). Yeast complementation experiments to verify the sugar transport functions of the ZosmaSWEETs gene family and found that *ZosmaSWEET8* can transport fructose, glucose, and sucrose (**Figure 7A**). This indicates the key role of *ZosmaSWEET8* in regulating sugar transport. Molecular docking results revealed the specific binding sites of ZosmaSWEET8 with substrates (glucose, fructose, sucrose). For instance, Asn176 and Asn196 within the ZosmaSWEET8 sequence specifically bind to glucose via hydrogen bonding interactions. By understanding the core binding sites between ZosmaSWEET8 proteins and their substrates (glucose, fructose, sucrose), a basis is provided for elucidating the molecular mechanism of sugar transport. SWEET proteins do not perform sugar transport functions independently, rather, they work synergistically in physiological processes such as transmembrane sugar transport by forming complexes with other proteins or regulating networks. Therefore, when screening the interacting proteins of ZosmaSWEET8, it was found that A0A0K9P0S4 had the highest connectivity which is a phosphoethanolamine N-methyltransferase that may methylate ZosmaSWEET8, thereby regulating its sugar transport activity. Based on this clear “transporter-substrate” correspondence, it is hypothesized that increasing the expression level of *ZosmaSWEET8* through regulatory approaches could enhance the transmembrane transport efficiency of glucose in *Zostera marina* cells, thereby promoting the targeted intracellular accumulation of glucose. Furthermore, as an important monosaccharide precursor for fucoidan biosynthesis (Chen, 2025), elevated intracellular glucose levels may provide a greater substrate supply for fucoidan assembly and thereby potentially promote fucoidan accumulation. However, the feasibility of this regulatory pathway remains to be verified through *in vivo* functional validation, glucose metabolic flux analysis, and systematic characterization of fucoidan content and structure, and the underlying molecular mechanisms still require further clarification. These findings outline a preliminary regulatory framework for ZosmaSWEET8-mediated sugar transport and provide important clues for subsequent *in vitro* validation and mechanistic investigations. More broadly, this work offers valuable insight into the molecular basis of sugar accumulation in the marine medicinal plant *Zostera marina* and provides a basis for future studies aimed at the sustainable development and high-value utilization of marine medicinal resources.

## 5 Conclusion

In this study, we systematically investigated *Zostera marina* from the perspectives of quality evaluation and functional gene analysis to support its resource development and quality control as a marine medicinal plant. Based on the determination of 11 soluble sugar components in four Zosteraceae species and subsequent chemometric analysis, *Zostera marina* was identified as a superior medicinal germplasm resource, and a soluble sugar-based quality evaluation system was established for Zosteraceae species. In parallel, eight SWEET sugar transporter genes were identified from the *Zostera marina* genome for the first time. Bioinformatic and phylogenetic analyses revealed that these genes are evolutionarily conserved and may be regulated by light and hormone-related signals. Functional assays further demonstrated that ZosmaSWEET8 mediates the transport of glucose, fructose, and sucrose, highlighting its potential role in sugar allocation and metabolism. Together, these findings establish a preliminary connection between soluble sugar-related quality traits and sugar transporter gene function in *Zostera marina*, and provide a foundation for future studies on germplasm improvement, molecular breeding, and the utilization of marine medicinal plant resources.

## Data Availability

The raw data supporting the conclusions of this article will be made available by the authors, without undue reservation.
