# Supplementary material for: Soluble sugar component-based quality assessment and SWEET transport family identification uncovering soluble sugar accumulation mechanism in Zostera marina
Source: Front Plant Sci. 2026 May 5;17:1789397. doi: 10.3389/fpls.2026.1789397 (PMC13185493; doi:10.3389/fpls.2026.1789397)
Supplement: Supplementary file 1 [file DataSheet1.pdf]

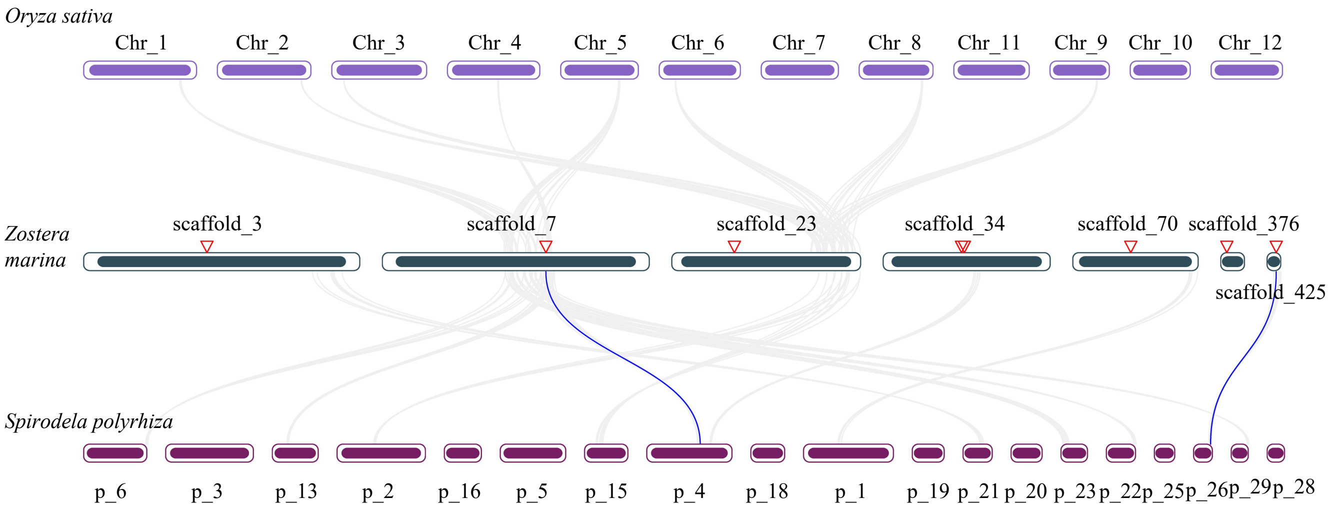

Figure S1. Based on genomic data, a collinearity analysis was conducted among *Oryza sativa*, *Zostera marina*, and *Spirodela polyrhiza*.

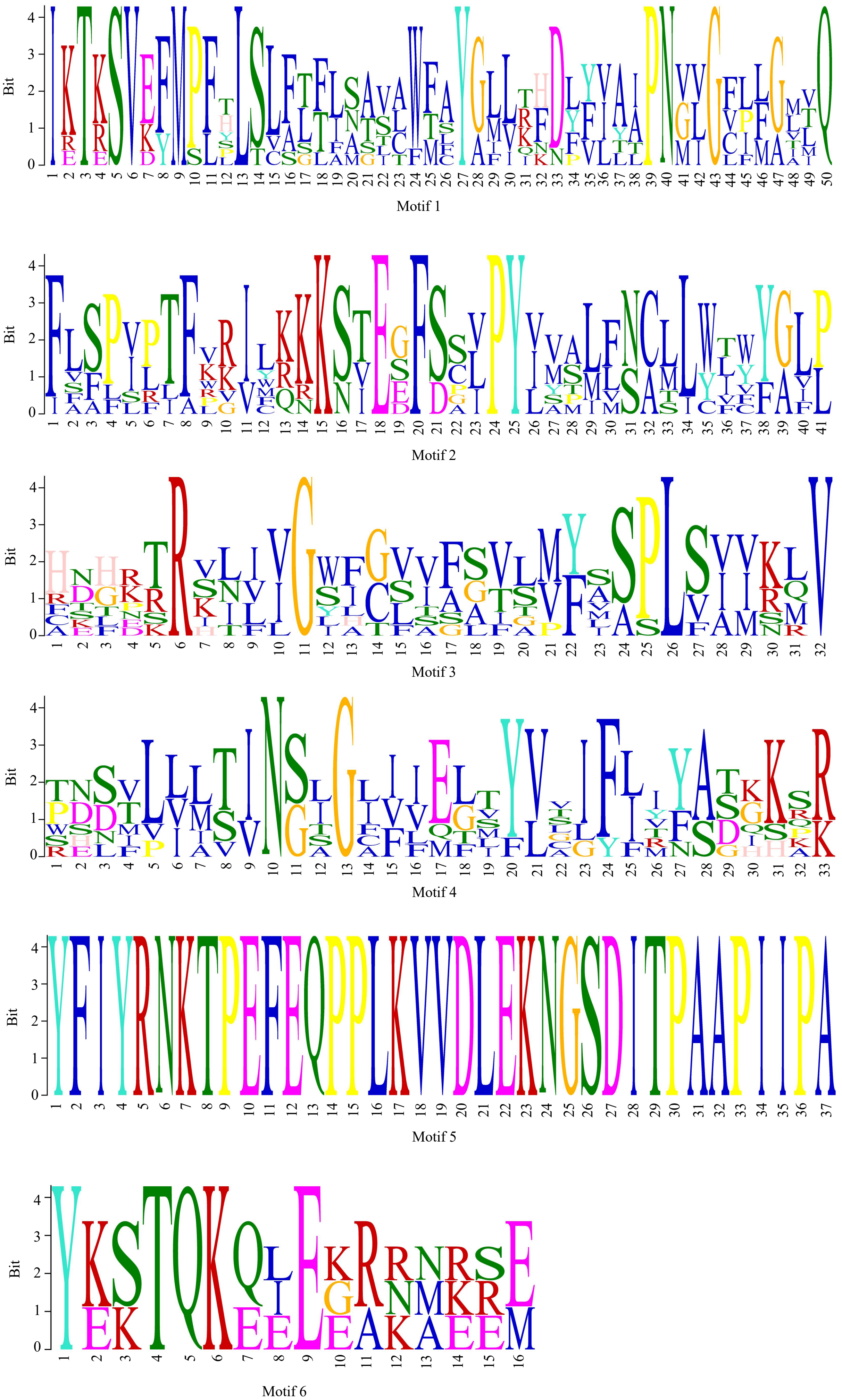

Figure S2. Conserved motifs of ZosmaSWEET proteins. In the sequence logo, each position within the motif is represented by amino acid letters. The horizontal axis indicates the position in the motif, and the height of each letter reflects the relative frequency of the corresponding amino acid at that position multiplied by the total information content of the stack.

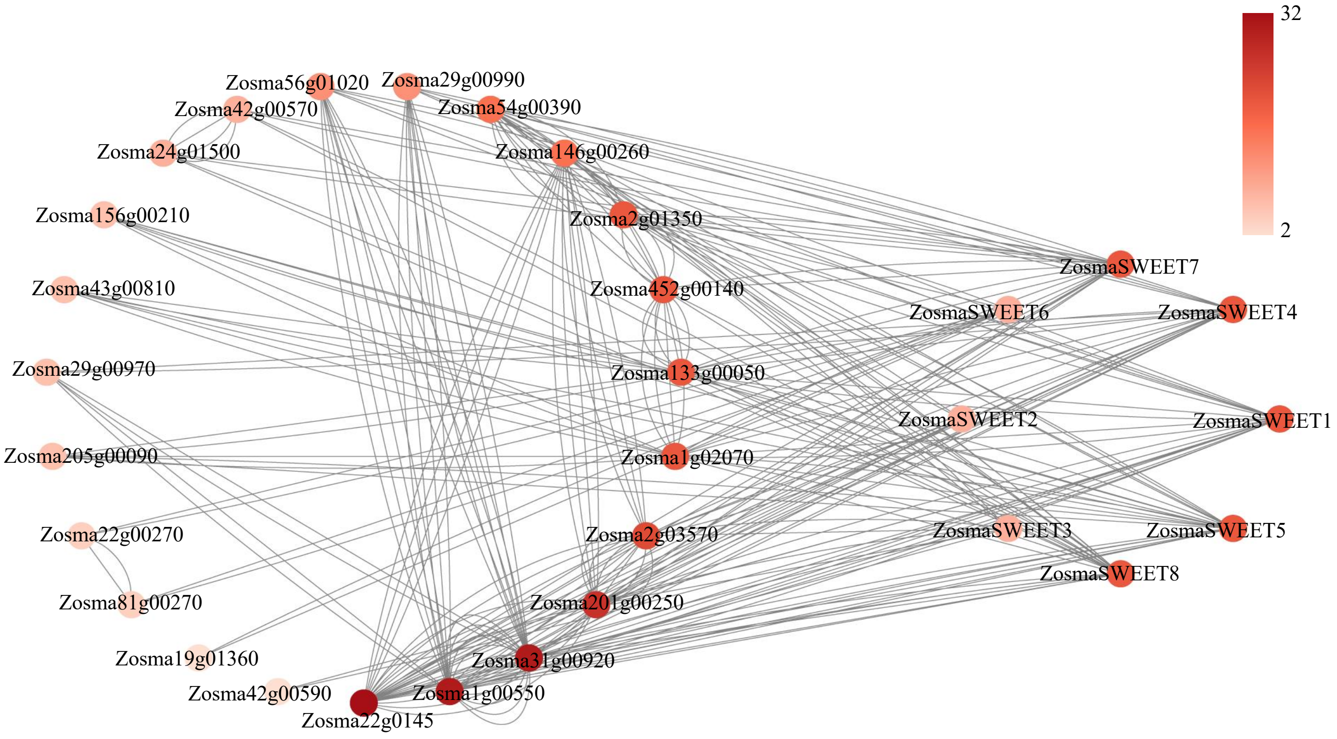

Figure S3. Visualization network of ZosmaSWEETs and interacting proteins. The darkness of the color indicates the number of interacting proteins.

Table S1 Ramachandran plot evaluation of AlphaFold-predicted ZosmaSWEET protein structures

Gene ID	AlphaFold ID	Core (%)	Allowed (%)	Generously allowed (%)	Disallowed (%)
ZosmaSWEET1	A0A0K9P605	93.2	4.2	1.8	0.8
ZosmaSWEET2	A0A0K9NMZ9	90.5	8.0	0.4	1.1
ZosmaSWEET3	A0A0K9PHD	97.3	2.2	0.4	0
ZosmaSWEET4	A0A0K9P6Z0	90.3	7.9	1.2	0.6
ZosmaSWEET5	A0A0K9P6Z0	90.3	7.9	1.2	0.6
ZosmaSWEET6	A0A0K9NQI0	96.2	3.3	0.5	0
ZosmaSWEET7	A0A0K9P5L8	96.6	3	0.5	0
ZosmaSWEET8	A0A0K9P282	96.5	3.1	0.4	0

Table S2 Primers used in this study

	Gene ID		Primer
Yeast complementation experiment	ZosmaSWEET1	Forward	tcccccgggctgcaggaattcATGAATCCATGGATCTTCGCTAC
		Reverse	gggcccccccctcgaggtcgacTTAGTTGCCAGAGCTTCCTTGAA
	ZosmaSWEET2	Forward	tcccccgggctgcaggaattcATGGTAGGTCTAGACCCCCGA
		Reverse	gggcccccccctcgaggtcgacTTACGGCATGGTTGGGACA
	ZosmaSWEET3	Forward	tcccccgggctgcaggaattcATGGCGATCGGCGTCG
		Reverse	gggcccccccctcgaggtcgacTCAAACAATACTATTTATTACATAATCTTGCA
	ZosmaSWEET4	Forward	agtggatcccccgggctgcagATGAACAACGTTGATTACTTTTGCA
		Reverse	gggcccccccctcgaggtcgacTCAAGCTGGAATTATTGGTGCA
	ZosmaSWEET5	Forward	agtggatcccccgggctgcagATGGGCGAGGCAAATCTCC
		Reverse	gggcccccccctcgaggtcgacTCAAGCTGGAATTATTGGTGCA
	ZosmaSWEET6	Forward	agtggatcccccgggctgcagATGGAGAAGTCAGACATTCATCAGT
		Reverse	gggcccccccctcgaggtcgacTCATGCATGCAGGTGCAGC
	ZosmaSWEET7	Forward	agtggatcccccgggctgcagATGGTTTCCCCCGATACTATCA
		Reverse	gggcccccccctcgaggtcgacTCATGTCATCTGGATGTTATTCTCG
	ZosmaSWEET8	Forward	tcccccgggctgcaggaattcATGATTTCAGCAGACACCGCA
		Reverse	gggcccccccctcgaggtcgacTTATGATCTCTGTTTAGGAGGATATCCG
